# Some Points in the Ether-Chloroform Controversy

**Published:** 1897-09

**Authors:** W. M. Barton

**Affiliations:** Washington, D. C.


ARTICLE II.

### SOME POINTS IN THE ETHER-CHLOROFORM CONTROVERSY.

BY W. M. BARTON, M. D., WASHINGTON, D. C.

Read before the Washington City Dental Society, June 15, 1897.

The subject of anesthesia has become so highly specialized that it is not at all an easy task to select a particular phase of it which might be generally interesting. Being fully aware of your indulgence, however, I have written upon a topic which interests me more than any question bearing upon the present status of anesthesia, namely, the controversy which is taking place to-day in all medical societies of the world between the adherents of ether on the one hand and those of chloroform on the other.

This subject, I take it, may be of interest to you from a practical as well as scientific point of view.

The cataphoric anesthetization of dental pulp by special solutions, as developed by Grasheintz, or infiltration of the cellular tissue of the gum along the lines mapped out by Schleich, are of interest to you as practicing dental surgeons, but the subject with which we are dealing is of greater moment to us all, as involving questions of ethics is of great importance.

It would be interesting certainly, had we time to enter into it, to discuss the question of discovery of general anesthesia by ether, for the question of priority of this discovery gave rise to one of the bitterest contests for fame ever waged in a medical arena.

Last year, 1896, being the fiftieth anniversary of the event and of its announcement to the world, very naturally the old discussion, temporarily crushed by the death of the principal combatants, were reopened. Papers by Barber and Emerson appeared in favor of Jackson, and a very calm and dispassionate argument, though brief, by Ayer, in favor of the claims of Morton. Whatever the real truth may be, the dentists and doctors of to-day may well join in congratulating themselves and in carrying the two names, with that of Long, emblazoned upon the shield of fame.

The question of discovery may well be dismissed with the following bulletin:

March 30, 1842, Crawford Long, of Georgia, used ether for surgical anesthesia. He repeated it four times between that and 1843 and then gave it up and made no public mention of it until 1849, three years after its general adoption.

In November, 1844, Horace Wells, of Boston, a dentist, making regular use of nitrous oxide for extraction, used ether instead, at the suggestion of Dr. Marcy, of Hartford, Conn. He soon abandoned it and went back to nitrous oxide.

In September, 1846, Charles T. Jackson, of Plymouth, Mass., suggested to Dr. William T. J. Morton the use of ether in place of nitrous oxide. Dr. Morton made the introduction of ether his life work.

The discovery soon crossed the Atlantic, and anesthesia by ether was taken up enthusically by Liston in England and by Malgaigne in France. The discovery produced the profoundest stir everywhere as such a revolutionary and wonderful procedure might have been supposed to do. The only objections urged against ether at this time were on moral grounds. A death occurred in England, and about the same time Sir James G. Simpson, of Scotland, introduced chloroform, and all of the enthusiasm which had before belonged to ether, and its promoters, for some unaccountable reason, went over to chloroform, which agent was used universally, and absolutely without discretion. Ether promised to be forgotten. Is it any wonder that all the enthusiastic rivalry as to who had used chloroform the greatest number of times should have been suddenly checked by a series of shocking accidents? Deaths occurred—unaccountable, sudden, unexpected, death in persons apparently sound, under short operation and early in the anesthetic state, when accidents might have been supposed least liable to occur. Physiologists and surgeons discovered simultaneously that they were handling a fanged serpent, whose venom was deadly, and who had come to them in the divine shape of Morpheus himself. Warm discussions arose between partisans everywhere, and France became divided. The Lyonnaise took up ether, the Parisians chloroform. America and England became divided in the same manner, while Germany and Belgium remained faithful to chloroform. These distinctions have remained to the present day and the discussions have remained, but the close observer can see that ether is gaining ground, and if there be anything in signs, chloroform is fast losing the enviable position of vantage which it has held for nearly a half century.

The more the question is studied the more do the partisans of ether multiply, and it looks as though the death knell of chloroform as a general anesthetic is about to be sounded.

If we could hope in the very brief limits of this paper, to treat the subject in an exhaustive manner, we would naturally adopt the classification adopted by that great therapeutist, H. C. Wood, in a paper read before the tenth International Medical Congress in 1890. He presented his essay under several points, the most important being: first, a discussion of the method by which these two drugs kill, both in man and the lower animals; that is to say, whether they destroy life through respiration or the circulation; second, a comparison of the comparative fatality, to which may be added a description of the phenomena observed upon administration of the two anesthetics. Some such order as this is a great convenience in treating this subject.

Unfortunately as regards the first proposition no gratuitous assumption can be made, as there are great difference of opinion, at least until very recently, as to the actual manner, for instance, by which chloroform produces death in ordinary cases. Two great schools have arisen upon this question arrayed in two lines, the one adhering to the cardia paralytic theory, the other to the respiratory-paralytic.

It is certainly very important to resolve this question, for unless we do we can form no precise and logical notion of the matter at all. This very point gave birth to the great Hyderabad commission, whose work on the subject has formed the inspiration, if not the basis, for most subsequent investigations by Gashell, Shire and McWilliams in England, and by Hare, Wood and Thornton in our own country.

The number of experiments conducted under the above auspices alone has been enormous.

Through the beneficence of the Nizam of Hyderabad, India, two commissions were appointed. The first has been justly criticised because it concluded that chloroform deaths are respiratory. A second commission was appointed by Lauder Brunton and came to identical conclusions, although Brunton when he came to India to conduct the investigation believed in the cardiac failure theory.

About this time Wood and Hare published a joint paper, in which they adhered to the powerful depression effect of chloroform on the heart, and McWilliams of Glasgow did the same. The Nizam of Hyderabad, bent upon pursuing the controversy to its bitter end, commissioned one of our own great therapeutists, Hobart Amory Hare, of Philadelphia, and Gaskell and Shire, of England, to pursue studies upon the subject, with a view of effecting a reconcilation. The result has been to emphasize the depressing effect of chloroform on the circulation and respiration, but more particularly to demonstrate the important fact *depression* and *paralysis* of the *vaso motor centres in the medulla* constitute the most important feature of the baneful effect of chloroform. In other words, it is the dilitation of capillaries, as well as enfeebleness of the heart, that we have to fear in administering chloroform, death being due to vaso-motor paralysis, literally allowing the patient to bleed to death in his own capillary system. The capillary area of the body is so comparatively enormous that it is capable of containing all the blood in the body, and if the heart cavities are not supplied with blood it is a well-known fact that that muscle will cease to functionate although it be not primarily diseased. Both ether and chloroform produce a fall in blood pressure, but the latter much more so than the former. This being the case, the chloroform advocates say bandage the extremities, give atropine, which is a vaso-motor stimulant, apply abdominal compression, invert the patient, &c., all these things tending theoretically to counterbalance the evil effects of chloroform, but practically they do not confer immunity from danger, and only give a false sense of security.

All of the conclusions, then, of the Hyderabad commission can by no means be accepted, and several objections can be urged, according to Wilson, against the increased application of this conclusion. This commissiom advocated chloroform because in some 600 administrations upon lower animals it was not found possible to directly paralyse the heart by ordinary doses. But there may, and probably does, exist a considerable difference between the effects of chloroform upon man and animals; the habits of life, the taking of stimulants and narcotics, certainly alter the resistance of the central nervous system. There are important differences in vital capacity between man and animals, the latter breathing less of the vapor than man.

It is a fact that when there is some obstruction to breathing in cases submitted to chloroform anesthesia, deaths are extremely rare. Clover, some time ago, called attention to the comparative immunity of phthisical patients, whose respiratory capacity is of course diminished. This same comparative immunity seems to exist in patients anesthetized on the side and in cases of ovarian tumors, under all of which circumstances less vapor enters the lungs. In this connection it is important to note, as Wilson has done, that the majority of deaths occur in robust persons who take chloroform eagerly for reduction of dislocations, dental extractions, and particularly when the anesthetic is administered in the upright position. For ordinary cases the several conditions giving a fatal result from heart failure are combined only about once in every two or three thousand cases, but because the commission did not obtain them in a few hundred experiments they unreasonably conclude that the danger does not exist.

All agree, then, that chloroform diminishes blood pressure, and the only question would seem to be whether it does so by a primary effect upon the heart muscle or by one upon the vaso-motor center. We have seen that recent investigations point to the latter theory.

Most of the anesthetists whom I have encountered who prefer chloroform to ether base their preference upon the fact that it is more easily handled; they say that the period of excitation is less marked and that the anesthetic state is more profound and less difficulty thereby encountered from struggling, spasm, and tremor. Chloroform *is* more easily handled in the sense of being carried, for it and its paraphernalia are less bulky, but the same amount of experience in manipulation of ether will soon convince the most incredulous of its great convenience and ease of administration, and these are the only questions which, in this particular discussion, should be admissible. It is very necessary to have a good apparatus to give ether on account of its great volatility, and since the spray apparatus of forty years ago many hundreds of cones have been invented. Of those which I have used, I prefer Clover's, but lately, in Europe, three inhalers have entirely displaced it—Julliard's, Chalots, and especially Wanscher's, which last bids fair to become the inhaler of the future. As regards the ether employed, it is of course important to have it pure, anhydrous, free from alcohol, boiling at 35° C. without odor or residue and without blue discoloration after addition of dilute sulphuric acid.

General anesthesia with ether ought to be obtained within six or seven minutes certainly, occasionally in two or three minutes, except in alcoholics. It requires, according to Angelesco, about 150 c.c. of ether for one hour of anesthesia with a closed inhaler, 200 c.c. for two hours and 300 c.c. for three hours. A man requires about one-third more than a woman. The slow method of administration is altogether to be preferred to the rapid, massive, and asphyxiating variety. The aspect of the patient anesthetized by ether is characteristic. There is first a mild period of excitation, increased only when the ether is given too concentrated from the first, but which is altogether insignificant in the vast majority of cases.

At the same time there is a slight cough, often absent if the vapor is not strong. The face becomes a little congested and more so if all air is excluded, in which case even cyanosis may intervene. In proportion as the anesthesia becomes more complete the respirations become regular, profound, and noisy, the last due to mucus in the bronchi, and all combined forming a guide as to the degree of anesthesia. The conjunctival reflex is lost. This aspect does not change with ether during the anesthesia. After the operation the effects last according to the length of the anesthesia. If prolonged one-half hour the effects last ten to twenty minutes, if two, to two and one-half hours, sleep may last one-half hour or even more, but never do we note that shock and prostration so often seen after chloroform. On waking there may be some excitement and mucus secretion; sometimes a slight chill, particularly in the extremities. Thirst and headache are fairly common, and lumbar pains, with occasional albuminuria. The contrary exists with chloroform, the period of excitation often wanting with ether is present usually. If it is troublesome with ether it is due to mal-administration. A cough occurs in 4 per cent. of the cases of ether anesthesia. Facial congestion is present in 90 per cent. or more, but it is not dangerous. Cyanosis should be avoided by careful attention to administration. Salivation is present in about 95 per cent. of the cases. It varies greatly in amount, under some circumstances continuing notwithstanding constant care, while at other times it may hardly be said to exist. It does not amount to much unless it becomes tracheobronchial, in which event it slightly impedes respiration. Ether may aggravate pulmonary diseases, if they exist, but does not seem to produce them any easier than chloroform. Vomiting is much less frequent with ether than with chloroform, when it is pure, and when the patient has not eaten on the day of operation. It is altogether rare for a chloroform patient not to vomit either during or after the administration of the anesthetic. Not so with ether. Julliard gives only one case of vomiting in twelve administrations; others, one in thirteen or one in fifteen. After ether it is common to have a little mucus regurgitated, but it is not violently projected. Vomiting does not last, as often occurs with chloroform, for hours after its administration. The respirations are very typical with ether, are always regular and attended with sounds audiable at a distance. They become more rapid during the first quarter of an hour of anesthesia, remain at one figure during its maintenance, and gradually descend. They rarely get over forty-five per minute. The pulse is strong and regular and will remain so, often for two hours. The maximum is about 115 per minute. This rapidity is not annoying because the pulse is strong, but with chloroform the pulse is often 140, and weak besides. The temperature declines notably under ether in the first hour, 2° to 2½°. It is due partly to evaporation, immobility, slowing of oxidations, but we do not know why ether produces greater reduction of temperature than chloroform. The pupils dilate before anesthesia is profound and then contract and remain so. Upon awakening the pupils dilate again.

A good many objectors to ether say that it causes nephritis. On animals, experiments were long ago made which pointed to the comparative innocuity of ether as regards the kidneys. Ether is less irritating to the kidneys according to unanimous consent of all experimentors. As to post anesthetic albuminuria, in 116 etherization, Angelesco found fourteen times a transient albuminuria disappearing in three or four days, and my own investigation at Columbia Hospital, in the gynælogical service of Drs. I. S. Stone and J. W. Bovee, showed figures approximately similar.

Let us sum up in a word some of the great practical advantages of ether.

1. The excitement stage is rarely seen except in alcoholics, and is more violent if the anesthetic is hurried.

2. All the time the patient is kept under ether a sonorous sound is heard, contrary to what passes with chloroform, where respiration is silent, so much so that the ear may have to be placed close to the chest to hear respiration. This snoring is the tranquility of the surgeon; his ear perceives it and he is not preoccupied with the condition of the patient. He does not have to stop to ask "if all is well;" "if the patient breathes well," &c.

3. If the anesthetic is given in too large doses and is accumulating the snore is transformed into a deep rhoncus, grave, and less regular, the face becomes cyanotic, the eye is congested. It suffices to remove the mask and to pull out the tongue and all is well. But there never appears that pallid tint, that cadaveric look which we see preceding a chloroform syncope.

4. The pulse, very active at first, soon slows, becomes regular, and is always strong and vibratory, contrary to chloroform narcosis, in which is rapid and soft.

5. The heart-beats are strong. Here is the indisputable advantage of ether. The etherized never have that terrible, sudden syncope, coming like a thunder-bolt out of a clear sky, followed by death, notwithstanding all we can do.

6. The accidents with ether are purely respiratory, slow and progressive, and consequently they leave us time to act.

7. The operation terminated, the patient may recover perfectly in one-fourth to one-half of an hour of his normal animation and color; there is none of that adynamia which we observe with chloroform. As Poncet has said, "the chloroformed are in a state of apparent death, the etherized in a state of profound intoxication."

8. Vomiting is certainly less frequent during and after ether. Of course a good deal of mucous comes up, but the patient can often eat an ordinary meal on the same evening.

Such is the comparison. Ether stimulates the circulation and heart, so it is especially precious in reduced patients. It is certainly less dangerous than chloroform, not only because the figures show this to be the case, but because the latter attacks the bulb and produces vasomotor paralysis and deleterious effects far beyond our control to limit. In ether the danger is more respiratory; it is slow and we can intervene, often successfully.

Let us look for a moment at the comparative statistics.

The Compte-Julliard-Gould tables contain 638,461 administrations of chloroform, with 170 deaths; 300,157 administrations of ether, with 18 deaths; giving chloroform one death in 3,749, and ether 1 death in 16,675. In the proceedings of the German Surgical Society, 1891, 65 European surgeons reported 23,000 cases of chloroform with 6 deaths, 1 in 3.776. At the 24th Congress of German Surgeons, 1895, statistics for 5 years showed for chloroform 1 death in 2,909.

American practice shows ether 1 death in 6,004.

The Hyderabad commission placed the mortality of chloroform to ether at about 2 to 1.

So also do the statistics of St. Bartholomew's Hospital, more favorable to chloroform than most statistics, place the relative mortality of chloroform to ether in the ratio of 4½ to 1.

Wood said in his Berlin address: "I doubt very much whether one-third of the deaths from anesthesia are reported; certainly not one-third of the cases I had personal knowledge of have been publicly recorded; moreover, the pressure to conceal deaths from chloroform is greater than when the lethal dose is due to ether. The surgeon who uses ether feels that he employed the safest anesthetic and that he will receive no blame if a death occurs from it and he feels, also, that he has a rare case to put on record, which will give his own name a prominent place in anesthetic literature. Whereas, a surgeon who uses chloroform knows that if death occurs from the anesthetic, a large proportion of the profession, at least in the United States, will condemn him, either in public or private, for the use of this drug, and that he will be fortunate if he escapes being publicly condemned by a coroner's jury. Moreover, deaths from chloroform are only too common, so that the surgeon has nothing to gain and much to lose by publication of a chloroform death, and, if possessed of the average human nature, holds his peace.

Let me close this rather desultory account by quoting the phlegmatic and metaphorical opinion of Alfonso

Aguilar, which I read recently in a Spanish journal. This gentleman prefers chloroform and ends up an article by stating that the only advantage of ether over chloroform is that any one can administer the former but not so the latter. With chloroform he says the patient ought to be kept well asleep and the anesthetizer attentive and wakeful, while during etherization they can both go to sleep without inconvenience.—*Dental News.*

